# Two-year real-world effectiveness of faricimab after treatment switch in neovascular age-related macular degeneration

**DOI:** 10.1186/s40942-026-00916-0

**Published:** 2026-08-04

**Authors:** Jorge Ruiz-Medrano, Margarita Zamorano, Carlota Moreno de Alboran, José M. Ruiz-Moreno

**Affiliations:** 1 Puerta de Hierro-Majadahonda University Hospital, C/Manuel de Falla 1, Madrid, 28222 Spain; 2 Department of Ophthalmology, Universidad Autónoma de Madrid, Madrid, Spain; 3 Department of Ophthalmology, Castilla La Mancha University, Albacete, Spain

**Keywords:** Faricimab, Neovascular age related macular degeneration, anti-VEGF injections

## Abstract

**Purpose:**

To evaluate the 2-year real-world outcomes of switching to intravitreal faricimab in patients with neovascular age-related macular degeneration (nAMD) previously treated with anti-VEGF agents and unable to extend treatment intervals (TI) beyond 12 weeks.

**Methods:**

Prospective cohort study including patients with nAMD managed under a treat-and-extend regimen who required frequent anti-VEGF injections (< 12-week intervals) to maintain retinal dryness. Patients were switched to faricimab without a loading phase and followed for a minimum of 2 years. TI were extended in 4-week increments based on anatomical response. The primary outcome was change in TIl; secondary outcomes included change in best-corrected visual acuity (BCVA) and identification of predictors of response. “Optimal responders” were defined as eyes achieving ≥ 8-week interval extension.

**Results:**

A total of 110 eyes from 97 patients were included (mean age 78.7 ± 9.8 years). Prior to switching, the mean TI was 6.18 ± 2.05 weeks, which increased significantly to 13.61 ± 5.62 weeks at the last follow-up visit (*p* < 0.001). BCVA remained stable over 2 years (0.42 ± 0.24 vs. 0.43 ± 0.25; *p* = 0.0117). At final follow-up, 68.2% of eyes achieved intervals ≥ 12 weeks, 20.0% ≥16 weeks, and 24.6% reached ≥ 20-week intervals. Overall, 89.1% of eyes experienced interval extension. Optimal responders accounted for 47.3% of eyes and were significantly younger and had received fewer prior injections (*p* < 0.05).

**Conclusions:**

Switching to faricimab in previous treated nAMD patients enabled meaningful extension of TI over 2 years while maintaining stable visual acuity. These findings support faricimab as an effective strategy to reduce treatment burden in patients who are difficult to extend with prior anti-VEGF therapies.

## Introduction

Age-related macular degeneration (AMD) is the leading cause of visual impairment and blindness across Europe. [1] Management is commonly based on repeated intravitreal injections (IVIs) of vascular endothelial growth factor (VEGF) inhibitors, which have reduced the risk of blindness associated with this condition. [2] Nevertheless, this treatment approach is associated with a considerable burden for patients, healthcare systems, and caregivers. [3, 4].

In 2022, the U.S. Food and Drug Administration (FDA) approved faricimab for the treatment of neovascular AMD (nAMD) following pivotal trials TENAYA and LUCERNE, which demonstrated that faricimab was noninferior to aflibercept 2 mg in terms of visual outcomes. [5, 6] Faricimab is a bispecific humanized monoclonal antibody that simultaneously targets and inhibits two key mediators of angiogenesis, VEGF A and angiopoietin-2 (Ang-2). [7] Dual inhibition of VEGF-A and Ang-2 is thought to enhance vascular stability while reducing neovascularization and inflammation, potentially leading to greater treatment durability compared with anti-VEGF monotherapy. [8].

Recent real-world studies have shown that faricimab can extend treatment intervals (TI) in patients with nAMD while maintaining stable visual and anatomical outcomes over 1 year. [9–16] However, evidence beyond 12 months remains scarce, with no published real-world data currently available evaluating outcomes at 2 years.

The aim of this study was to evaluate, in a real-world clinical setting, the ability of faricimab to extend TI over a 2-year follow-up in patients with neovascular age-related macular degeneration (nAMD) who were previously treated with other anti-VEGF agents and were unable to achieve dosing intervals beyond 12 weeks.

## Methods

The study was approved by the Ethics Committee of Puerta de Hierro-Majadahonda University Hospital (protocol number 31/25) and followed the tenets of the Declaration of Helsinki, International Council for Harmonization (ICH) guidelines, Good Clinical Practice (GCP) standards, and applicable Spanish laws. To ensure patient confidentiality, all identifying data were either encrypted or appropriately anonymized.

### Study participants

The treatment protocol has been reported previously.⁹ Briefly, this prospective cohort study enrolled consecutive patients aged ≥ 18 years with nAMD receiving anti-VEGF therapy under a treat-and-extend (T&E) regimen, whose TI could not be extended beyond 12 weeks while maintaining retinal dryness, and who had a minimum follow-up of 2 years. Anatomical stability before switching to faricimab was defined as the maintenance of a dry macula under the previous anti-VEGF regimen, characterized by the absence of intraretinal fluid (IRF), subretinal fluid (SRF), and other signs of active exudation on spectral-domain optical coherence tomography (SD-OCT) (Heidelberg Spectralis or Topcon SS Triton). Pigment epithelial detachment was not considered an exclusion criterion unless associated with signs of active neovascular activity or fluid accumulation.

Patients were offered a therapeutic switch to faricimab to assess the potential for further extension of TI. Following the switch, patients received an initial faricimab injection at the same interval as prior to switching. Subsequent intervals were extended in 4-week increments only if the retina remained dry. Retinal dryness was assessed qualitatively on SD OCT at each visit and defined as the absence of intraretinal or subretinal fluid. In cases where both eyes met inclusion criteria, both were included in the analysis.

All participants underwent a complete ophthalmic evaluation, including decimal best-corrected visual acuity (BCVA), slit-lamp examination of the anterior segment, intraocular pressure measurement by Goldmann applanation tonometry, and dilated fundus examination using indirect ophthalmoscopy. Multimodal imaging was also performed, consisting of color fundus photography and SD-OCT. When both eyes were eligible, all assessments were conducted bilaterally.

### Study outcomes

The primary endpoint was the TI between faricimab IVIs. The secondary endpoint was the change in decimal BCVA from baseline (initial interval) to the last follow-up visit (LFUV). Baseline characteristics were studied in order to establish their potential influence in patients’ response to the switch of treatment including: type of CNV, accumulated number of previous IVIs, previous anti-VEGF agents used, initial BCVA, baseline TI, age, gender and laterality.

As in our previously published 1-year analysis [9], “optimal responders” were defined as eyes achieving a TI extension of ≥ 8 weeks. This threshold was chosen because it represents a clinically meaningful improvement in treatment durability and a substantial reduction in treatment burden in patients who had been unable to extend beyond 12 weeks before switching to faricimab. Eyes that did not achieve an interval extension of ≥ 8 weeks were classified as “non-optimal responders”.

### Statistical analysis

Statistical analysis was performed using IBM SPSS Statistics software (version 29.0.2.0; Chicago, IL, USA). Continuous variables were reported as mean ± standard deviation (SD), and categorical variables as counts and percentages. Normality of data distribution was assessed using the Kolmogorov–Smirnov test. Differences in TI and BCVA were analyzed using the paired Student’s t-test, while comparisons of quantitative variables between optimal and non-optimal responders were performed using the independent Student’s t-test. Categorical variables were compared with the chi-square test. A p-value < 0.05 was considered statistically significant.

## Results

**Fig. 1:**
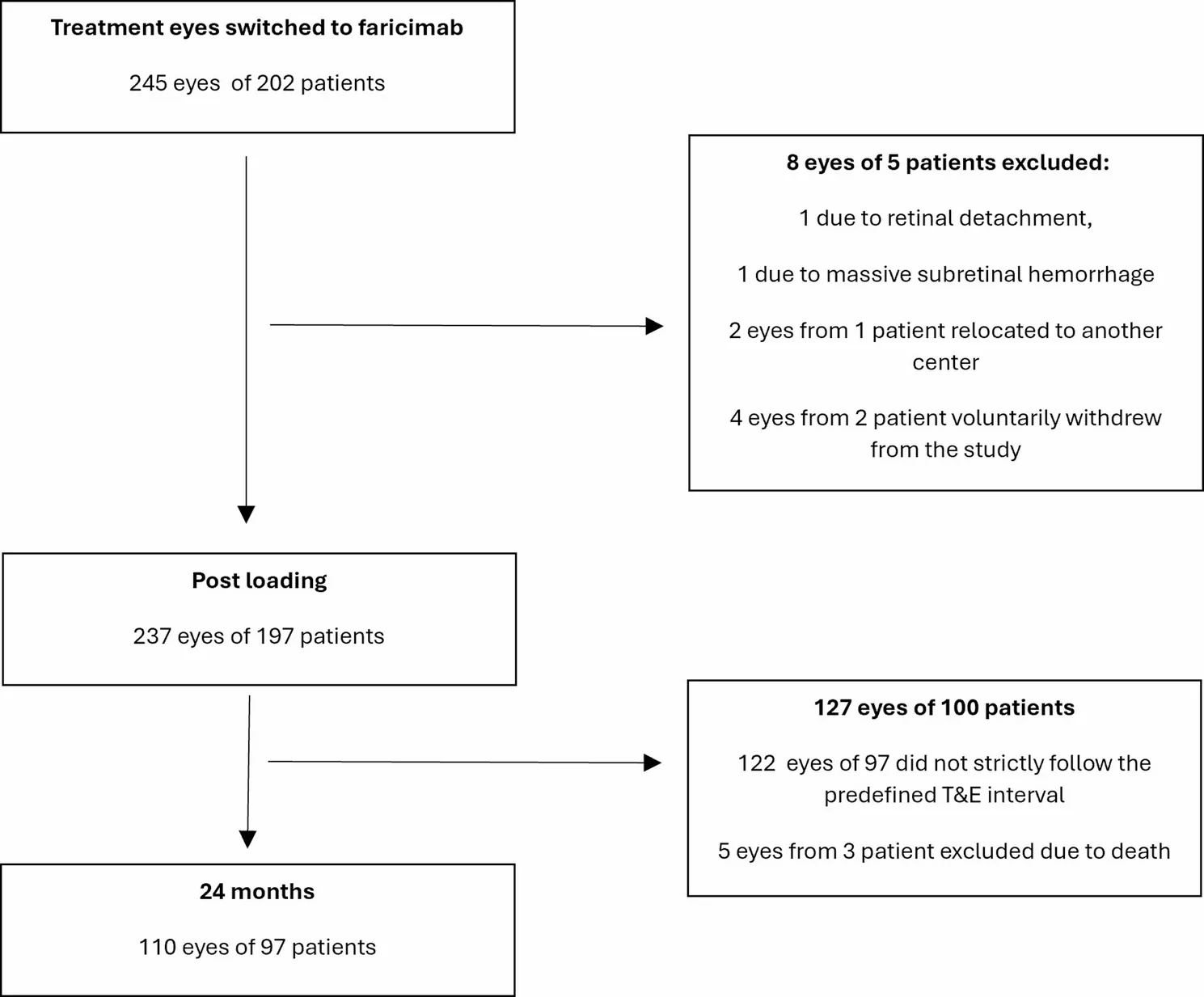
Flowchart diagram showing the inclusion, exclusion, and follow-up of eyes after switching treatment to faricimab

A total of 110 eyes from 97 patients who met the inclusion criteria and had a minimum follow-up of two years after switching to faricimab were analyzed. The number of patients lost to follow-up and reasons for treatment discontinuation are shown in Fig. 1. A considerable number of eyes were excluded from the 24-month analysis because strict predefined T&E criteria were applied. Eyes that did not follow the scheduled treatment interval pathway (Q8, Q12, Q16, and Q20) were excluded to ensure a consistent and rigorous assessment of treatment durability. A tolerance window of ± 1 week around each predefined treatment interval was established. Eyes treated outside this predefined window were excluded from the analysis because interval extension could not be consistently attributed to the predefined treatment algorithm. This criterion was established before the analysis to maintain methodological consistency in evaluating treatment durability.

The mean age of the participants was 78.7 ± 9.84 years, ranging from 69 to 94. Among the 97 patients, 59 (53.6%) were women, and 62 (56.4%) had their left eye included in the study. Prior to enrollment, 11.8% patients had been previously treated with ranibizumab, 66.4% with aflibercept, 1.8% with brolucizumab, and 20% with bevacizumab. 60.9% of eyes were diagnosed with type I CNV, 22.7%% with type II CNV, and 14.5% with type III CNV. Patients received a mean of 19.77 ± 15.06 anti-VEGF IVIs prior to switch. Decimal BCVA at baseline before switch was 0.42 ± 0.24. Baseline characteristics of the 97 patients were analyzed as shown in Table 1.

**Table 1:** Baseline characteristics of our study population

Age (years)	78.7 ± 9.84 (Range, 69–94)
Gender, n (%)	
• Male	51 (46.4%)
• Female	59 (53.6%)
Eye injected, n (%)	
• Right	48 (43.6%)
• Left	62 (56.4%)
Anti-VEGF used before switch, n (%)	
• Aflibercept 2 mg	73 (66.4%)
• Bevacizumab	22 (20%)
• Ranibizumab	13 (11.8%)
• Brolucizumab	2 (1.8%)
Type of choroidal neovascularization (CNV), n (%)	
• CNV Type I	67 (60.9%)
• CNV Type II	25 (22.7%)
• CNV Type III	16 (14.5%)
Number of anti-VEGF injections before switch	19.77 ± 15.06
Mean interval of last injections until switch (weeks)	6.18 ± 2
Visual acuity (decimal Snellen chart)	0.42 ± 0.24

**Fig. 2:**
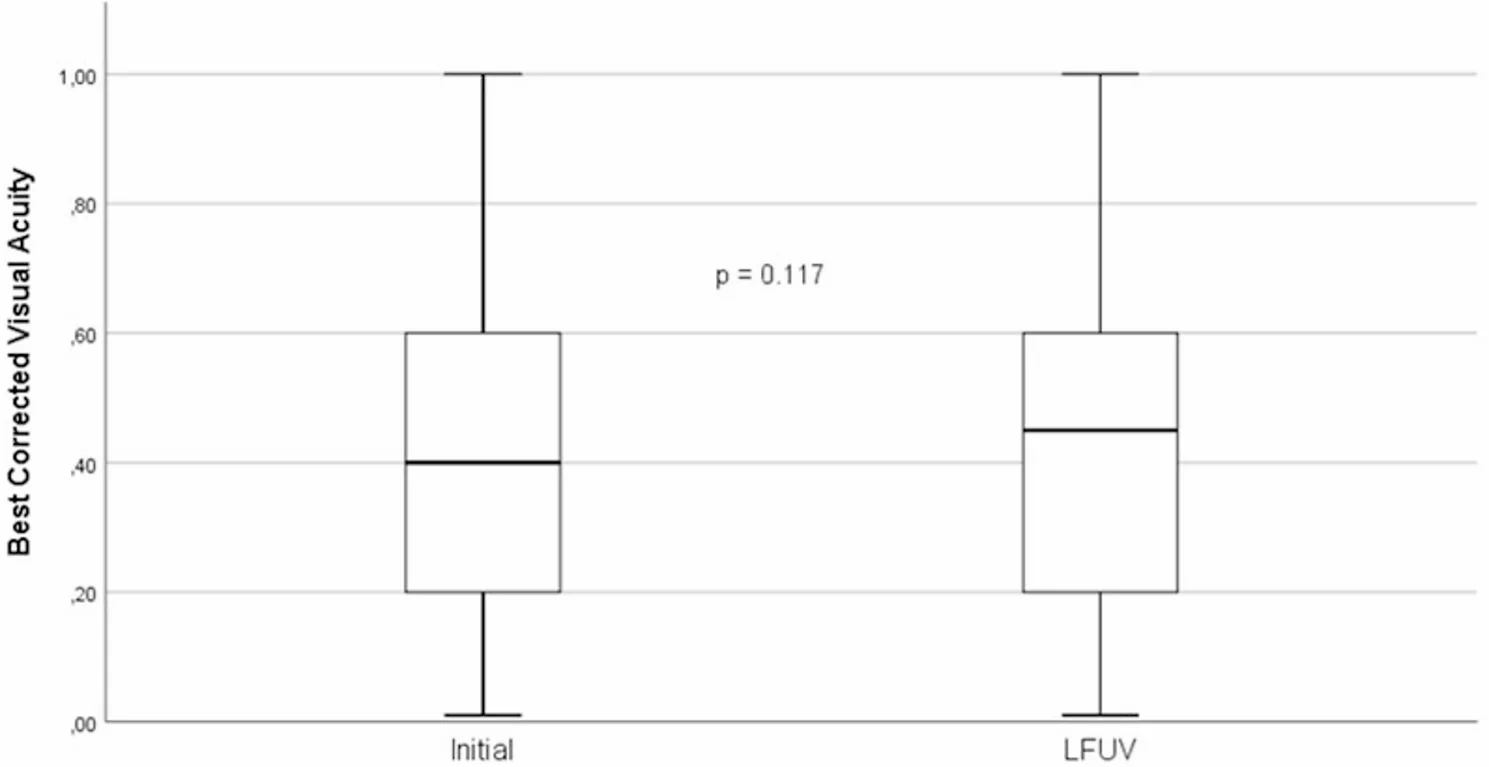
Box plot illustrating the absence of differences in best corrected visual acuity (BCVA) between the initial interval and the last follow-up visit (LFUV) after switching to intravitreal faricimab injections

The initial TI was established at 6.18 ± 2.0 weeks (range 4–10 weeks), corresponding to the interval before switching to faricimab. After the switch to faricimab, a statistically significant difference was observed between the mean TI and the final follow-up interval, which increased to 13.61 ± 5.62 weeks (range 4–24 weeks; *n* = 110; *p* < 0.001, paired Student’s t-test). Extension of TI was permitted only in cases of complete anatomical response, defined as the absence of intra- or subretinal fluid. Meanwhile, BCVA remained stable increasing from 0.42 ± 0.24 to 0.43 ± 0.25, with no statistically significant difference to report (*p* = 0.117; paired Student’s t-test) (Fig. 2).

**Fig. 3:**
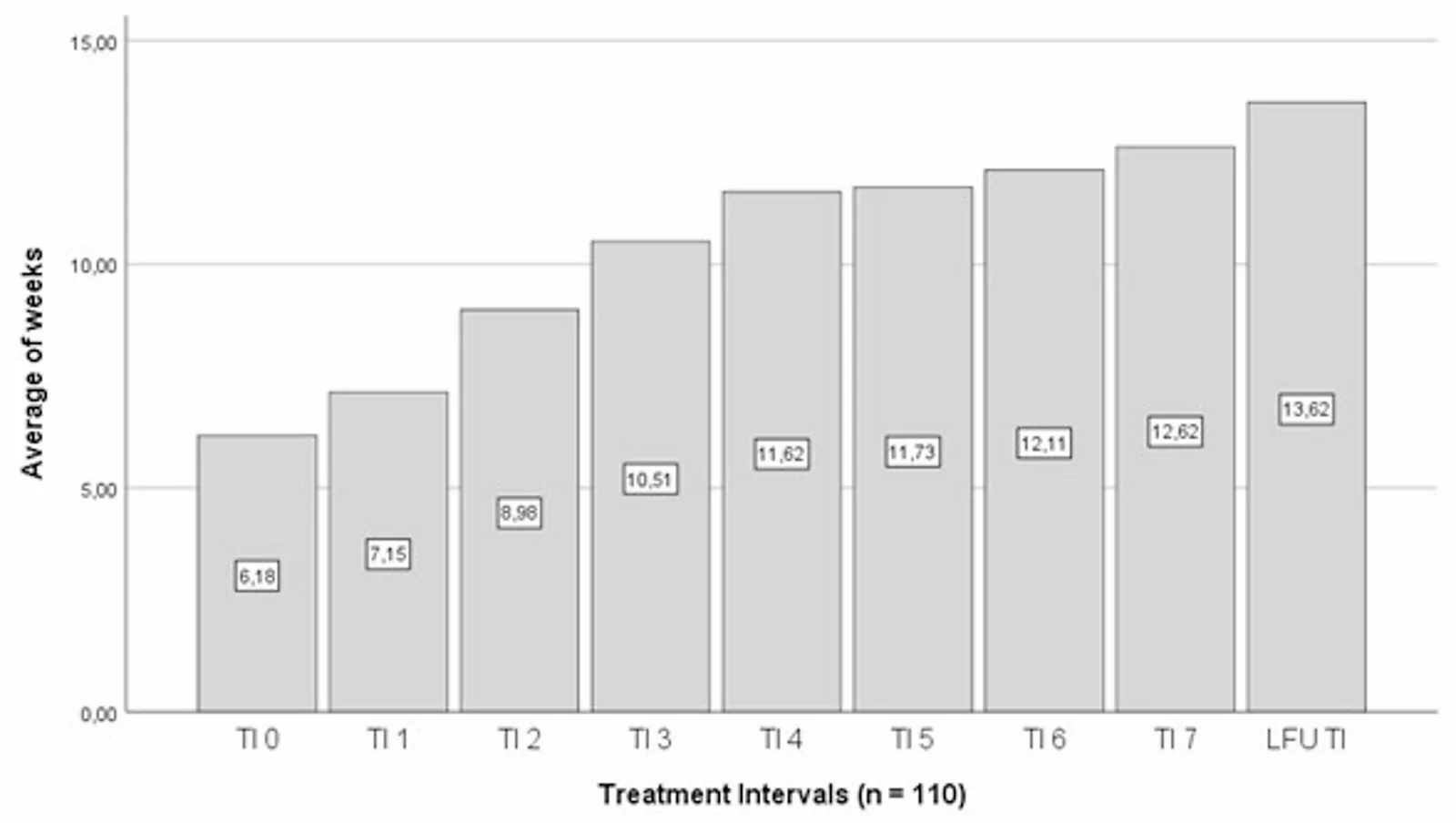
Comparison of the distribution of treatment intervals with different anti-VEGF agents at baseline and at the last follow-up visit after switching to intravitreal faricimab. Differences were statistically significant (*p* < 0.0001, chi-square test)

The distribution of TI changed significantly over time. At baseline, 100% (110 eyes) were treated at intervals of less than 12 weeks. At 12 months, 38 eyes (35.8%) remained on intervals of less than 12 weeks, whereas 72 eyes (67.8%) achieved intervals longer than 12 weeks, including 33 eyes (31.1%) with intervals of at least 16 weeks and 11 eyes (10.38%) with 20-week intervals. At 24 months, among the 110 eyes that completed the minimum 2-year follow-up, 35 eyes (31.8%) remained on intervals of less than 12 weeks, whereas 75 eyes (68.2%) achieved intervals longer than 12 weeks, including 22 eyes (20.0%) with intervals of at least 16 weeks and 27 eyes (24.6%) with 20-week intervals (*p* < 0.0001, chi-squared test) (Fig. 3).

Among the 110 eyes analyzed, 3 eyes (2.7%) required a shorter TI at the last follow-up compared with baseline. In 9 eyes (8.2%), the interval remained unchanged, as no further extension was feasible. Conversely, 98 eyes (89.1%) achieved an extension of the TI, indicating that the target was met in the majority of cases.

**Fig. 4:**
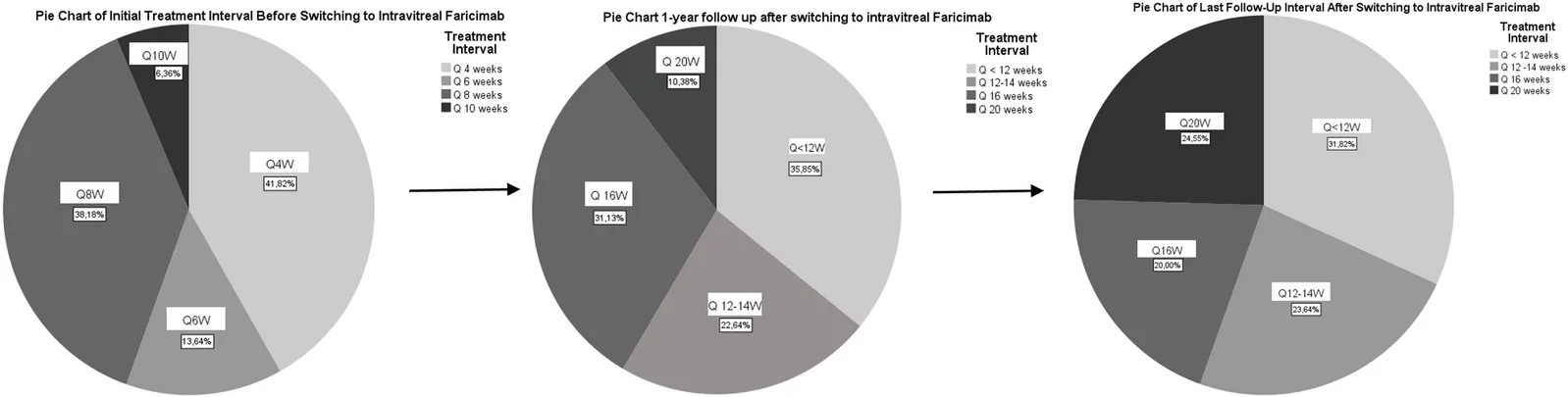
Mean treatment intervals after switch to faricimab during 2 year follow-up. (TI: Treatment Interval; LFU: Last follow-up)

The mean initial TI was set at 6.18 ± 2.05 weeks (range 4–10 weeks), corresponding to the interval prior to switching to faricimab. After the switch to faricimab, significant increases in the TI were observed at each subsequent time point compared with TI0 (*p* < 0.001 for each, respectively). These differences remained significant in the subsequent intervals (Fig. 4).

Patients received a mean of 19.77 ± 15.06 IVIs prior to switch and a mean of 11.20 ± 3.42 after faricimab introduction (*p* = 0.0117; paired Student’s t-test). The mean follow-up period was 116. 09 ± 10.08 weeks (range: 104–148 weeks).

We performed an additional analysis including all 232 eyes (the 110 eyes included in the per-protocol analysis plus the 122 eyes excluded because they did not strictly follow the predefined T&E schedule). In this analysis, the mean treatment interval increased significantly from 7.05 ± 2.8 weeks (range, 4–16) before switching to 11.90 ± 5.5 weeks (range, 4–24) at the last follow-up (*p* < 0.001, paired Student’s *t*-test).

A total of 58 eyes (52.7%) were classified as “non-optimal responders” and 52 (47.3%) as “optimal responders.” Optimal responders were defined as patients whose interval between IVIs was extended by more than 8 weeks. No statistically significant differences were observed between the two groups in terms of laterality, gender, CNV subtype, previous anti-VEGF treatment, initial BCVA, or baseline TI. In contrast, statistically significant differences were found when comparing age and the number of previous IVIs. (Table 2).

**Table 2:** Quantitative variables comparison between “optimal responders” and “non optimal responders”

	Non-Optimal Responder (*n* = 58)	Optimal Responder (*n* = 52)	*p*-value
BCVA	0.41 ± 0.22	0.42 ± 0.27	0.8
Age (years)	80.56 ± 5.91	76.54 ± 12.48	0.01*
Nº IVI	22.37 ± 16.42	17.67 ± 14.54	0.04*
Baseline Treatment Interval (weeks)	6.0 ± 1.96	6.33 ± 2.10	0.3

†Paired Student’s T-test. (BCVA: best corrected visual acuity, Nº IVI: number of intravitreal injections)

In terms of safety, no cases of retinal vasculitis, RPE tear, or infectious endophthalmitis were observed.

## Discusion

The high treatment burden associated with intravitreal anti-VEGF therapy remains a major challenge in the management of nAMD. This real-world study builds on our previously published 1-year results, in which switching to faricimab within a T&E regimen enabled significant extension of TI while maintaining stable BCVA in patients with nAMD. The current analysis extends these findings to a 2-year follow-up, providing additional evidence on the durability and long-term effectiveness of faricimab in routine clinical practice. In this study, retinal monitoring was performed using either spectral-domain OCT (Heidelberg Spectralis) or swept-source OCT (Topcon DRI OCT Triton), according to routine clinical practice. Although the use of different OCT platforms may introduce some variability in image acquisition and retinal thickness measurements, treatment decisions were based on qualitative assessment of disease activity rather than automated quantitative parameters. Both devices allowed adequate evaluation of relevant exudative biomarkers, including intraretinal fluid, subretinal fluid, pigment epithelial detachment, and other signs of neovascular activity, which were used to guide treatment interval adjustments. The initial treatment interval was 6.18 ± 2.0 weeks (range 4–10 weeks), corresponding to the interval before switching to faricimab. After the switch, a sustained and statistically significant interval extension in was observed, increasing to 13.61 ± 5.62 weeks at the end of follow-up (range 4–24 weeks; *n* = 110; *p* < 0.001, paired Student’s t-test). Notably, this extension over time was achieved together with stable visual outcomes, reinforcing the consistency of our initial observations and supporting the long-term benefit of this therapeutic strategy in a population with high treatment burden and limited response to prior anti-VEGF agents.

To evaluate the potential impact of the exclusion criterion on selection bias and the generalizability of our findings, we performed a sensitivity analysis including all eyes, regardless of the strict predefined T&E schedule. Although the mean treatment intervals differed slightly from those of the per-protocol cohort (baseline: 7.05 vs. 6.18 weeks; last follow-up: 11.90 vs. 13.61 weeks), the improvement in treatment interval remained highly statistically significant (*p* < 0.001). These findings did not materially influence the primary study outcome, as treatment intervals remained significantly longer after switching to faricimab even when all eyes were included in the analysis.

Our results are consistent with the 2-year outcomes of the TENAYA and LUCERNE trials, [6] including the Japan subgroup, [17] where the visual and anatomical benefits achieved at year 1 were sustained through year 2, with a high proportion of patients maintained on extended dosing intervals of up to 16 weeks. In that context, more than 60% of patients achieved Q16W dosing at week 112, highlighting the durability of dual Ang-2 and VEGF-A inhibition. More recently, post hoc analyses from TENAYA/LUCERNE have suggested that over 50% of patients may be eligible for further extension to 20-week dosing intervals under a treat-and-extend approach, reinforcing the potential for even greater durability with faricimab [18].

In parallel, our real-world data demonstrate that similar durability can be achieved in previously treated patients, with significant extension of treatment intervals and stable visual outcomes over 2 years. Notably, a meaningful proportion of eyes in our cohort reached treatment intervals of ≥ 20 weeks, supporting the feasibility of these extended regimens outside clinical trial settings. Importantly, unlike clinical trial populations, our cohort consisted of chronic, previously treated patients with limited interval extension prior to switching, which may better reflect routine clinical practice. Therefore, our findings complement pivotal trial data by confirming that the long-term benefits of faricimab can be translated into real-world settings, even in more challenging cases.

In our cohort, the proportion of eyes achieving extended treatment intervals remained high at year 2. Compared with the 1-year follow-up, a greater proportion of patients at 2 years received faricimab every 12 weeks (31.82% vs. 22.7%), whereas fewer were treated every 16 weeks (20.0% vs. 35.1%) and more every 20 weeks (24.55% vs. 4.0%). The proportion of patients requiring treatment intervals shorter than 12 weeks at year 2 was similar to that observed at 1 year (31.82% vs. 38.2%). The sustained increase in dosing intervals could reflect improved vascular stability mediated by Ang-2 inhibition. Furthermore, half of the cohort met the criteria to be labelled as “optimal responders,” defined as an increase of interval extension of ≥ 8 weeks. Interestingly, no significant differences were observed between optimal and non-optimal responders in terms of baseline BCVA, CNV subtype, prior treatment, or baseline treatment interval. However, younger age and a lower number of previous intravitreal injections were associated with a better response. Optimal responders were younger and had received fewer previous intravitreal injections, suggesting that a lower cumulative treatment burden and potentially less advanced disease chronicity may be associated with greater treatment durability after switching to faricimab. Conversely, patients with a longer treatment history and higher number of previous injections may represent a more refractory subgroup with greater disease chronicity and reduced potential for interval extensión. Nevertheless, these observations should be interpreted with caution, as this study was not designed to identify predictive factors of response, and the relatively limited sample size prevents definitive conclusions regarding baseline characteristics associated with optimal response. Further studies are required to determine whether demographic and treatment-related factors can predict long-term treatment durability with faricimab.

Collectively, real-world studies with 1-year follow-up have consistently shown that switching to faricimab in treatment-experienced nAMD patients allows extension of treatment intervals while maintaining stable visual outcomes. Ambati et al. [10] and Goodchild et al.[11] reported significant interval prolongation with preserved BCVA, while Kataoka et al.[12] and Grimaldi et al.[13] found that approximately 40% of eyes can achieve extended dosing schedules in previously treated populations. Qaseem et al.[14] and Cancian et al. [15] also described anatomical improvement with variable degrees of fluid resolution in refractory cases. In the study by Sim et al.,[16] treatment intervals increased from 4.2 ± 0.3 weeks pre-switch to 6.9 ± 2.3 weeks at 12 months, although the reduction in injection burden was modest (0.9 fewer injections), likely related to the use of a loading-dose phase prior to interval extension, which delayed early individualization of dosing.

In contrast, our protocol did not include a post-switch loading phase. Instead, patients received an initial injection at their pre-switch treatment interval, allowing confirmation of a dry retina before initiating extension. Treatment intervals were then progressively individualized without reintroducing loading regimens, reflecting a more flexible real-world strategy that may have facilitated an efficient interval prolongation while maintaining stable visual acuity over 2 years.

Importantly, our 2-year real-world data extends previous evidence by demonstrating that these gains in treatment interval durability and visual stability are maintained over a longer follow-up than previously reported, thereby strengthening and expanding the evidence base for faricimab in pretreated nAMD. To our knowledge, no real-world studies have yet reported 2-year outcomes, which precludes direct comparison with our findings.

This study has several limitations that should be acknowledged. First, although prospective, it was conducted at a single center, which may affect the generalizability of the results. Second, The absence of a control group is an important limitation, as it prevents direct comparison with patients continuing their previous anti-VEGF therapy and precludes definitive conclusions regarding the superiority of faricimab. However, the aim of this study was to evaluate the potential benefit of switching to faricimab in a real-world population with a high treatment burden and limited capacity for interval extension under previous therapy. Therefore, our findings should be interpreted as evidence of treatment durability and feasibility in clinical practice rather than as proof of comparative efficacy. Third, Variability in prior treatments and disease duration may have influenced treatment response. Fourth, the limited number of eyes analyzed may prevent the extrapolation of these results. In addition, quantitative anatomical outcomes, including central retinal thickness and longitudinal changes in intraretinal or subretinal fluid, were not systematically collected for the present analysis. Retinal dryness was assessed qualitatively on SD-OCT and was used as the criterion for TI extension. Importantly, all included eyes were anatomically stable under their previous anti-VEGF regimen; however, treatment intervals could not be extended beyond a certain threshold while maintaining that stability, which was the rationale for switching to faricimab. Although the inclusion of eyes with a dry retina may raise the possibility of selection bias, this population reflects a clinically relevant subgroup of patients with a high treatment burden despite controlled disease activity. Nevertheless, the real-world design, the prospective follow-up, and the inclusion of a substantial cohort of chronic, previously treated eyes represent important strengths of the study and provide clinically meaningful insights into everyday practice.

In conclusion, switching to faricimab in previously treated nAMD patients resulted in significant extension of treatment intervals while maintaining stable visual acuity over 2 years. These findings support the role of faricimab as an effective and safe option to reduce treatment burden in routine clinical practice. Further studies with larger cohorts and comparative designs will be needed to confirm these results and to better identify predictors of optimal response.

## Data Availability

The data supporting the findings of this study are available from the corresponding author upon reasonable request.
